# Prompt response to rituximab in a patient with resistant polymyositis with complications of dysphagia and dysphonia: a case report

**DOI:** 10.1097/MS9.0000000000002490

**Published:** 2024-08-22

**Authors:** Yousef Mahmoud Nimer Habes, Saif Ibraheem Mohammad Sadeh, Layth Jamil Mohammad Saada, Ali Jamil Ali Mali, Mohammed Abdulrazzak, Saed I. Y. Attawna

**Affiliations:** a Faculty of Medicine, Al-Quds University, Jerusalem; b Department of Internal Medicine, Al-Ahli Hospital, West Bank, Palestine; c Faculty of Medicine, University of Aleppo, Aleppo, Syria

**Keywords:** case report, dysphagia, polymyositis, rituximab

## Abstract

**Introduction and importance:**

Polymyositis is an inflammatory process, primarily affecting proximal muscles, characterized by elevated muscle enzymes and distinctive electromyography patterns.

**Case presentation:**

The authors present a case of a 33-year-old male patient experiencing complications of polymyositis, including pharyngeal and laryngeal involvement leading to dysphagia and dysphonia. Steroids and intravenous immunoglobulin (IVIG) therapy proved ineffective. Subsequently, rituximab was administered, resulting in significant improvement in dysphagia, dysphonia, and proximal muscles within 3 days of the initial rituximab dose. Additionally, there was a remarkable decrease in creatine phosphokinase (CPK) levels.

**Clinical discussion:**

Immune-mediated myopathies (IMM) are rare diseases characterized by muscle inflammation and weakness. This case of probable polymyositis, diagnosed through clinical features and elevated CPK, was complicated by the patient’s lack of response to glucocorticoids and IVIG therapy. Remarkably, rituximab treatment led to rapid improvement in muscle strength and symptoms, highlighting its potential effectiveness in refractory cases of polymyositis.

**Conclusions:**

Primary treatment for cases of polymyositis typically involves the use of glucocorticoids. However, approximately half of the patients do not respond adequately to corticosteroids alone. Alternatives, in such cases, encompass IVIG therapy and rituximab.

## Introduction and importance

Highlights

- A 33-year-old male patient, with no significant medical history, was referred to the medical ward due to a 6-month history of progressively developing symmetrical weakness in the upper and lower limbs.
- Considering the clinical context, positive anti-Jo-1 antibodies in laboratory tests, and EMG results, the suspicion of polymyositis arose.
- The patient received two doses of 1 gram per infusion intravenously, spaced 3 weeks apart.

Polymyositis is an inflammatory condition affecting the muscles, particularly the proximal muscles, characterized by elevated serum muscle enzymes and distinctive electromyography findings. A muscle biopsy reveals necrosis of muscle fibers and an endomysial mononuclear inflammatory infiltrate. The reported incidence of polymyositis ranges from 0.41 to 0.75 per 100 000 individuals per year^1^. A separate study estimated the prevalence of polymyositis and dermatomyositis in 2003 at 21.5 per 100 000, with higher rates among older individuals, women, and urban residents^2^.

In polymyositis, T-cell-mediated cytotoxic inflammation targets unidentified muscle antigens. Although the triggering factors are unclear, viruses, particularly the HIV, can induce the condition. Proximal muscle weakness is the predominant presenting feature, occurring in approximately 69% of patients, with a higher prevalence in children (91%)^3^.

Serum creatine kinase (CK) serves as the most sensitive laboratory marker of muscle destruction. CK levels are consistently elevated in uncontrolled polymyositis. Clinicians should be alerted to the possibility of inclusion body myositis if CK levels are normal or mildly elevated (less than ten times normal)^4^.

The optimal initial treatment for polymyositis involves corticosteroids. For patients with resistant disease who do not respond adequately to glucocorticoids plus either azathioprine or methotrexate, alternative treatments such as rituximab, cyclosporin, intravenous immunoglobulin (IVIG), tacrolimus, or mycophenolate mofetil can be considered. Factors associated with a poorer outcome and reduced response to treatment include older age, greater weakness at presentation, underlying malignancy, pulmonary involvement, dysphagia, and delays in diagnosis or treatment initiation^5^.

The current paper presents a case of a 33-year-old male patient was referred to the medical ward due to a 6-month history of progressively developing symmetrical weakness who was suspected of polymyositis arose according to the clinical context, positive anti-Jo-1 antibodies in laboratory tests, and EMG results, providing valuable insights into the neurological development and highlighting the diagnostic challenges associated with such cases.

## Case presentation

A 33-year-old male patient, with no significant medical history, was referred to the medical ward due to a 6-month history of progressively developing symmetrical weakness in both the proximal and distal muscles of the upper and lower limbs. The weakness started gradually and increased steadily over time, not influenced by movement, and was accompanied by a sensation of feverishness. Notably, there was an absence of muscle pain and no weakness observed in the neck flexors. The patient reported no history of skin rash, arthralgia, myalgia, weight loss, or chronic drug use. Both past medical and surgical histories were unremarkable, with no known allergies to drugs or food. The family history was also unremarkable.

During the physical examination, the patient demonstrated cooperation, orientation to date, place, and person, and was afebrile. No signs of jaundice, cyanosis, or pallor were observed, and vital signs were within normal limits. Despite the patient appearing ill, there was no muscle or joint tenderness, and no limitations in movement were noted. Power in both upper limbs was graded as ⅘, while in both lower limbs, it was graded as ⅖, with intact sensation. Liver span was normal, and a general systemic review was unremarkable.

Laboratory tests revealed elevated levels of aspartate aminotransferase and alanine aminotransferase, both five times above normal limits. Additionally, the tests indicated a positive antinuclear antibody (ANA), a positive anti-histidyl transfer RNA [t-RNA] synthetase (Anti-Jo-1), and elevated creatine phosphokinase (CPK), with no other significant findings.

Laboratory tests are included in Tables 1 and 2. Abdominal ultrasound (US) and computerized tomography (CT) scans yielded no notable findings. A chest CT scan also revealed no abnormalities. Electromyography (EMG) displayed myopathic changes. Considering the clinical context, positive anti-Jo-1 antibodies in laboratory tests, and EMG results, the suspicion of polymyositis arose.

**Table 1:** Laboratory tests

Test	Result
Leukocyte count	18 480 WBCs/µl
Neutrophils	83.9%
Lymphocytes	10.8%
Monocytes	4.8%
Eosinophils	0.1%
Basophils	0.4%
C-reactive protein (CRP)	7.8 mg/l
Aspartate aminotransferase (AST)	163 U/l
Alanine aminotransferase (ALT)	272 uU/ml
Hepatitis B surface antigen (HBSAG)	Negative
Hepatitis C virus	Negative
ANA-Elisa antibodies	Positive
ds-DNA IgG	Negative
ds-DNA IgM	Negative
Total mitochondrial antibodies	Negative
Anti-smooth muscle antibodies	Negative
Antineutrophil cytoplasmic antibodies (C-ANCA-PR3)	Negative
Liver kidney microsome type 1 (LKM-1) antibodies	Negative
COOMBS test	Positive
CPK	2029 U/l
CK-MB	Elevated
troponin	Negative
Lactate dehydrogenase (LDH)	791 U/l
Urinalysis	Unremarkable

ANA, antinuclear antibody; CPK, creatine phosphokinase; WBC, white blood cell.

**Table 2:** Myositis-specific autoantibodies

Antibody	Result
Anti-Jo-1	Positive
Anti-U1-snRNP	Negative
Anti-SS-A	Negative
Anti-SS-B	Negative
Anti-scl-70	Negative
Anti-sm	Negative
Anti-snRNP/Sm	Negative
Anti-CenpB	Negative
Anti-HMGCR	Negative
Anti-SRP	Negative
Anti-Mi-2	Negative
Anti-MDA5	Negative
Anti-TIF1γ	Negative

Despite the administration of a high-dose steroid, the patient did not exhibit a significant response. After 1 month, muscle weakness worsened and became coupled with esophageal dysphagia and dysphonia, with no improvement in laboratory tests. Both the chest X-ray and CT scan showed no remarkable features.

The patient underwent a 5-day course of IVIG at a dosage of 400 mg/kg, resulting in minimal improvement in upper and lower proximal muscle weakness, while dysphagia and dysphonia remained unaffected.

Consequently, the decision was made to pursue rituximab therapy. The patient received two doses of 1 g per infusion intravenously, spaced 3 weeks apart. Remarkably, there was rapid and significant improvement in dysphagia, dysphonia, and upper and lower proximal muscle power within the initial four days. Subsequent serological assessments showed a considerable decrease in CPK to 409 U/l, along with the normalization of ALT, AST, and CRP levels. The patient was discharged on azathioprine. Follow-up over the next 2 months revealed no recurrence of previous symptoms, and serological results returned to normal.

## Discussion

The immune-mediated myopathies (IMM) are a rare and diverse group of diseases that have similar presentations of muscle inflammation and weakness. One member of this group is polymyositis (PM), which remains the most elusive one. Most of the time, a PM diagnosis is made by combining clinical features and laboratory findings^6^.

According to Bohan and Peter criteria^7,8^, the case is probable polymyositis as there was symmetric proximal muscle weakness, an increase in serum creatine phosphokinase CPK, and a characteristic EMG pattern with no rash, and no biopsy was done. According to the IIM calculator (score of 7.6) in the European League Against Rheumatism/American College of Rheumatology Classification Criteria (EULAR/ACR)^9^, the case is a definitive idiopathic inflammatory myopathy (IIM).

The ANA-Elisa and Anti-Jo-1 antibodies were found to be positive in autoantibody testing results. An observational retrospective cohort analysis of 260 patients with myositis revealed four clusters. Patients with dermatomyositis exhibited a rash that corresponded with positive Mi-2, anti-melanoma differentiation-associated protein 5 (MDA5), or anti-transcription intermediary factor-1 (TIF1) antibodies, which were all negative in our case. While anti-SRP or anti-3-hydroxy-3-methylglutaryl-coenzyme A reductase (HMGCR) antibodies were detected in individuals with immune-mediated necrotizing myopathy, both were negative in our case. Also, the presence of anti-Jo corresponds to anti-synthetase syndrome^10^. Positive anti-Jo-1, elevated CPK, and EMG findings with this clinical presentation correspond to polymyositis.

Glucocorticoids are considered a cornerstone initial therapy for IMM, despite the absence of placebo-controlled trials that show their effectiveness^11^. With glucocorticoid use, more than 80 percent of patients with IMM develop improvement. However, in PM, ~50% of patients don’t respond well to glucocorticoids alone^12^.

The patient was started on high-dose prednisone (1 mg/kg/day) for 6 weeks without side effects. So, the patient was referred for IVIG therapy, which was given for 5 days without clinical (proximal muscle weakness, dysphasia, and dysphonia) or laboratory improvement (CK, AST, ALT, and LDH).

However, a retrospective study (Marie *et al*.^13^) was made on 73 patients with PM and DM; all of them had steroid-refractory esophageal involvement, and 82.2% of all patients exhibited resolution of esophageal clinical manifestations by IVIG therapy. Another study (Saito *et al*.^14^) reviewed 15 patients with PM and DM who were resistant to steroid therapy; 100% of them showed improvement in CK level after 1 week of IVIG therapy, and 80% demonstrated improvement in daily activities after 2 weeks of IVIG therapy.

As there was no significant improvement after receiving both high-dose steroids and IVIG, the patient was started on rituximab therapy, as a study showed that it can be effective in patients with inflammatory myopathies who have refractory inflammatory myopathies during a median of 27.1 months of follow-up^15^. On the fourth day after receiving the first dosage of rituximab, the patient’s upper and lower proximal muscle power was restored by about 60%, and dysphagia and dysphonia symptoms improved dramatically. After 3 weeks, the second dose was administered, which resulted in a total of about 80% improvement in proximal muscle weakness as well as the cessation of dysphagia and dysphonia during the first three days of the second dose. It is rare for polymyositis not to respond to glucocorticoids. Even with responders to rituximab, the response will not be as rapid as in this case.

According to Aggarwall *et al*.^16^, the presence of an anti-synthetase [primarily anti-Jo-1], anti-Mi-2, or other myositis autoantibody indicated a shorter duration needed to improve in rituximab-treated refractory myositis patients, when compared to autoantibody-negative patients, who had a worse outcome. When our patient attended our clinic, he had a negative autoantibody basis [a negative ENA panel], which predicted a poor response to rituximab, despite the fact that his symptoms improved dramatically, markedly, and rapidly after rituximab treatment.

In considering factors influencing treatment response in idiopathic inflammatory myopathies (IIM), it is important to recognize that age is not the only determinant of therapeutic efficacy. While older patients are often associated with poorer outcomes due to various comorbidities and disease severity, in a retrospective cohort study, De Souza *et al*.^17^ reported that young idiopathic inflammatory myopathies (IIM) patients with dysphagia were more likely to be non-responders to rituximab, whereas our patient had a rapid and notable response to rituximab treatment. At the same time, CPK, LDH, liver enzymes, and other serology tests almost normalized after the treatment course. The patient was recommended to take Azathioprine 50 mg once daily and taper steroids. Follow-up during the next two months showed no relapse of any of the previous symptoms.

## Conclusions

While glucocorticoids are recognized as the primary initial treatment for immune-mediated myopathies, it’s noteworthy that nearly half of the patients do not exhibit a satisfactory response to corticosteroids alone. In such cases, alternative treatment options may involve IVIG therapy. Rituximab treatment is generally advised when there is a lack of response to prior interventions, particularly considering this patient’s rapid and impressive response to rituximab therapy.

## Ethical approval

Not applicable.

## Consent

Written informed consent was obtained from the patient and patient’s family for reporting this case and its associated images. The consent is available for review on request. All authors provide consent for publication.

## Source of funding

Not applicable.

## Author contribution

All authors fulfill the authorship criteria because of their substantial contributions to the conception, design, analysis, and interpretation of the data. Y.M.N.H.: wrote the abstract and introduction, reviewed the manuscript, and signed the consent form with the patient. S.I.M.S.: collected data about the patient and wrote the case presentation. L.J.M.S.: participated in data collection; participated in writing the discussion section. A.J.A.M.: participated in writing the discussion section and wrote the conclusion. M.A. reviewed the article, prepared the final submission, and submit the article. S.I.Y.A.: The attending physician, who treated the patient, reviewed the manuscript overall.

## Conflicts of interest disclosure

The authors declare no conflicts of interest.

## Research registration unique identifying number (UIN)

Our research study does not involve human subjects.

## Guarantor

Yousef Mahmoud Nimer Habes.

## Data availability statement

Not applicable.

## Provenance and peer review

Not commissioned, externally peer-reviewed.
